# A protocol for gene expression analysis of chondrocytes from bovine osteochondral plugs used for biotribological applications

**DOI:** 10.1016/j.mex.2017.10.005

**Published:** 2017-10-25

**Authors:** Christoph Bauer, Eugenia Niculescu-Morzsa, Stefan Nehrer

**Affiliations:** Center for Regenerative Medicine and Orthopedics, Department for Health Sciences and Biomedicine, Danube-University Krems, Dr.-Karl-Dorrek-Strasse 30, Krems, Austria

**Keywords:** Gene expression of bovine osteochondral plugs, RNA, Biotribology, Osteochondral plugs, Gene expression, Chondrocyte

## Abstract

RNA isolation from human or animal cartilage tissue is necessary when performing mechanical or biotribological applications. Despite no influence on the cells and no alterations in gene expression patterns, enzymatic digestion of tissues should be avoided as it’s known that the expression of collagen 2 can be effected (Hayman et al., 2006 [1]). After mechanical or biotribological tests alternative options with an immediate disruption of the tissue should be contemplated. To obtain RNA, different tissue homogenization and disruption methods are available on the market (Yu et al., 2004 [2]), but not everyone is suitable for cartilage. Some of them neither homogenize the cartilage, while others are producing a lot of foam during disruption process. After trying some of the currently available methods, we chose the MagNA Lyser Instrument from Roche to disrupt the cartilage and further isolate RNA by using the Fibrous Tissue Kit from Qiagen. After RNA isolation, cDNA synthesis was performed by additionally adding RNA from bacteriophage MS2 for stabilization purposes. For the RTqPCR bovine primers were designed and tested for efficiency to confirm that the whole gene expression analysis is working. Our protocol explains a whole method to perform gene expression analysis from bovine cartilage, but can also be used for human or any other animal tissue.

## Graphical abstract

**Figure:**
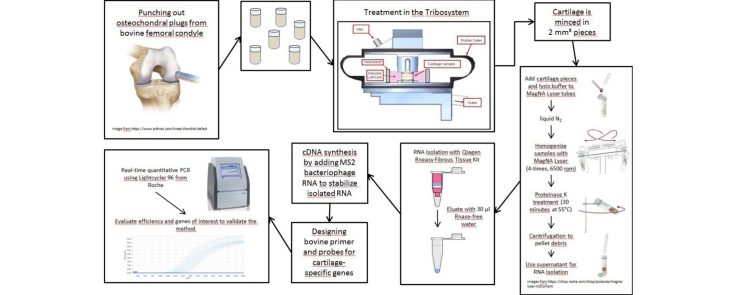

## Description of protocol

### Preparation of osteochondral plugs

1. Bovine knee was dissected with a scalpel to open the joint
2. Osteochondral (OC) plugs were punched out from the medial condyle from mostly flat areas
3. During unscrew from the punch, the OC plugs were marked at the bone to have the orientation of the collagen fibrils for further mechanical or biotribological testing
4. Each OC plug was put in a well of a 12-well plate and overlaid with PBS containing Antibiotics
5. After harvesting procedure, OC plugs in the 12-well plate were washed for 2 h in a shaker (Enviro-Genie; settings: 12:24; 37 °C) to remove fat and bone particles
6. Incubate the OC plugs for up to 1 week in growth medium with antibiotics, antimycotic and Vitamin C until further treatment in mechanical, biotribological or stimulation applications.
7. If transport of the OC plugs or the cartilage is required, conservation in RNAlater™ is possible for up to 1 week at 4 °C until RNA isolation

### RNA isolation

Note: for the main RNA isolation steps (we were using the protocol from Qiagen for the RNeasy Fibrous Tissue Kit)

1. After treatment or stimulation of the OC Plugs the cartilage is cut off from the bone and chopped to approximately 2 mm^3^ small pieces
2. Cartilage pieces are put in a MagNA Lyser tube with 300 μl Lysis Buffer (containing 1% β-mercaptoethanol added) from RNeasy Fibrous Tissue Kit
3. Freezing the samples in liquid nitrogen
4. Thaw the samples for 2 min and put them immediately into the MagNA Lyser, which is a suitable homogenization method [2], to disrupt the still frozen samples more efficiently without using enzymatic digestion [1]
5. Disrupting is done 4-times with 2 min of cooling (4 °C in a special cooling plate which comes with the MagNA Lyser device) after each run (settings: 6500 rpm for 20 s)
6. For every disrupted sample mix 20 μl of proteinase K with 580 μl of RNase-free water, add it to the MagNA Lyser tube and incubate this at 55 °C for 30 min
7. Centrifuge the samples for 3 min at 10,000*g* and transfer the supernatant to a new 1.5 ml tube
8. Add 0.5 volumes of 90–100% ethanol and mix gently
9. Transfer 700 μl of the sample to an RNeasy Mini column and centrifuge for 15 s at 8000*g*
10. Discard the flow through and repeat this step until complete lysate is used
11. Add 350 μl Buffer RW1 to the RNeasy Mini column and centrifuge for 15 s at 8000*g*
12. Discard the flow through, mix 10 μl DNase stock solution with 70 μl Buffer RDD and add this to the RNeasy Mini column membrane
13. Incubate for 15 min at room temperature (20–30 °C)
14. Add 350 μl Buffer RW1 to the RNeasy Mini column and centrifuge for 15 s at 8000*g*
15. Discard the flow through and add 500 μl Buffer RPE to the RNeasy Mini column and centrifuge for 15 s at 8000*g*
16. Discard the flow through and add 500 μl Buffer RPE to the RNeasy Mini column and centrifuge for 2 min at 8000*g*
17. Place the RNeasy column in a new 2 ml collection tube and centrifuge at full speed for 1 min
18. Place the RNeasy column in a new 1.5 ml tube, add 30 μl RNase-free water and centrifuge for 1 min at 8000*g*
19. Store the eluated RNA at −80 °C until cDNA synthesis

### cDNA synthesis

Note: We are using RNA from bacteriophage MS2 to stabilize the isolated RNA during cDNA synthesis with the Transcriptor First Strand cDNA Synthesis Kit

1. Thaw the reagents and mix them like in Table 1 (showing a single reaction)

**Table 1:** Reagents used in a single reaction for cDNA synthesis.

Reagent	Single reaction	Final concentration
Transcriptor RT Reactions Buffer 5× conc.	6.000 μl	8 mM MgCl
Protector RNase Inhibitor 40 U/μl	0.750 μl	50 U
Deoxynucleotide Mix 10 mM each	3.000 μl	1 mM
Random Hexamer Primer 600 μM	3.000 μl	60 μM
Transcriptor Reverse Transcriptase 20 U/μl	0.750 μl	25 U
Bacteriophage MS2 RNA 0.8 μg/μl	0.375 μl	10 μg/ml
Nuclease free distilled water	0.125 μl	
**Total volume**	**14.000** **μl**
2. Dispense the volume of a single reaction (14 μl) to each tube and add 16 μl of RNA sample to each reaction
3. Performing cDNA synthesis with the iCycler from BioRad in following steps
  a Primer annealing for 10 min at 25 °C
  b cDNA synthesis for 60 min at 50 °C
  c denaturation for 5 min at 85 °C
  d cooling phase for 5 min at 20 °C
4. cDNA is stored at −20 °C until RTqPCR for up to 1 month

### Real-time quantitative PCR (RTqPCR)

1. bovine primers and probes for the following genes were designed by using the RealTime qPCR software from IDT (Integrated DNA Technologies) on their website (https://eu.idtdna.com/site)
  a GAPDH (Glyceraldehyde 3-phosphate dehydrogenase)
  b COL2A1 (Collagen type 2)
  c ACAN (Aggrecan)
  d COL1A1 (Collagen type 1)
  e MMP-1 (Matrix Metalloproteinase-1)
  f MMP-13 (Matrix Metalloproteinase-13)
2. In Table 4 the design parameters of the bovine primers and probes for the relevant genes are shown
3. Synthesis of the bovine primers and probes was done from IDT
4. After receiving the primers and probes, the experimental determination of the annealing temperature was done by performing qPCR for a temperature gradient. Note: We use double-quenched probes, because they provide consistently lower background, resulting in higher signal compared to single-quenched probes [3]. All probes used a 5′ 6-FAM™ with an internal ZEN Quencher and 3′ Iowa Black^®^ Fluorescent Quencher.
5. As a next step, the evaluation of the efficiency was done. Note: Efficiency is an essential marker in real-time gene quantification procedure [4] and in our case, it is used to confirm, that the primers and probes are working and RTqPCR can be done for the specific genes of interest;
6. Reagents showed in Table 2 (showing a single reaction) are mixed to evaluate the efficiency as well as gene of interests

**Table 2:** Master mix used for RTqPCR used for a single reaction.

Reagent	Single reaction	Final concentration
FastStart Probe Master 2×	5 μl	1×
Hydrolysis Probe 2.5 μM	1 μl	0.25 μM
Left Primer 5.0 μM		0.50 μM
Right Primer 5.0 μM		0.50 μM
Nuclease free distilled water	3 μl	
**Total volume – Master Mix**	**9** **μl**
7. Dispense the master mix of a single reaction (9 μl) to each well of a 96-well PCR plate and add 1 μl of complementary DNA (cDNA) to each reaction
8. All tested conditions were performed in triplicates
9. After adding the master mix and cDNA to the 96-well PCR plate, the plate is closed with LightCycler^®^ 480 Sealing Foil and centrifuged for 10 min, 877*g* and 4 °C
10. RTqPCR was performed using the LightCycler^®^ 96 from Roche with following settings
  a Preincubation at 95 °C for 10 min
  b step amplification (repeated 45 times)
    i 95 °C for 10 s
    ii 65 °C for 30 s
  c Cooling at 37 °C for 30 s
11. After completion, the efficiency for the individual gene was calculated automatically (Fig. 1) by the LightCycler^®^ 96 SW 1.1 software

**Fig. 1:**
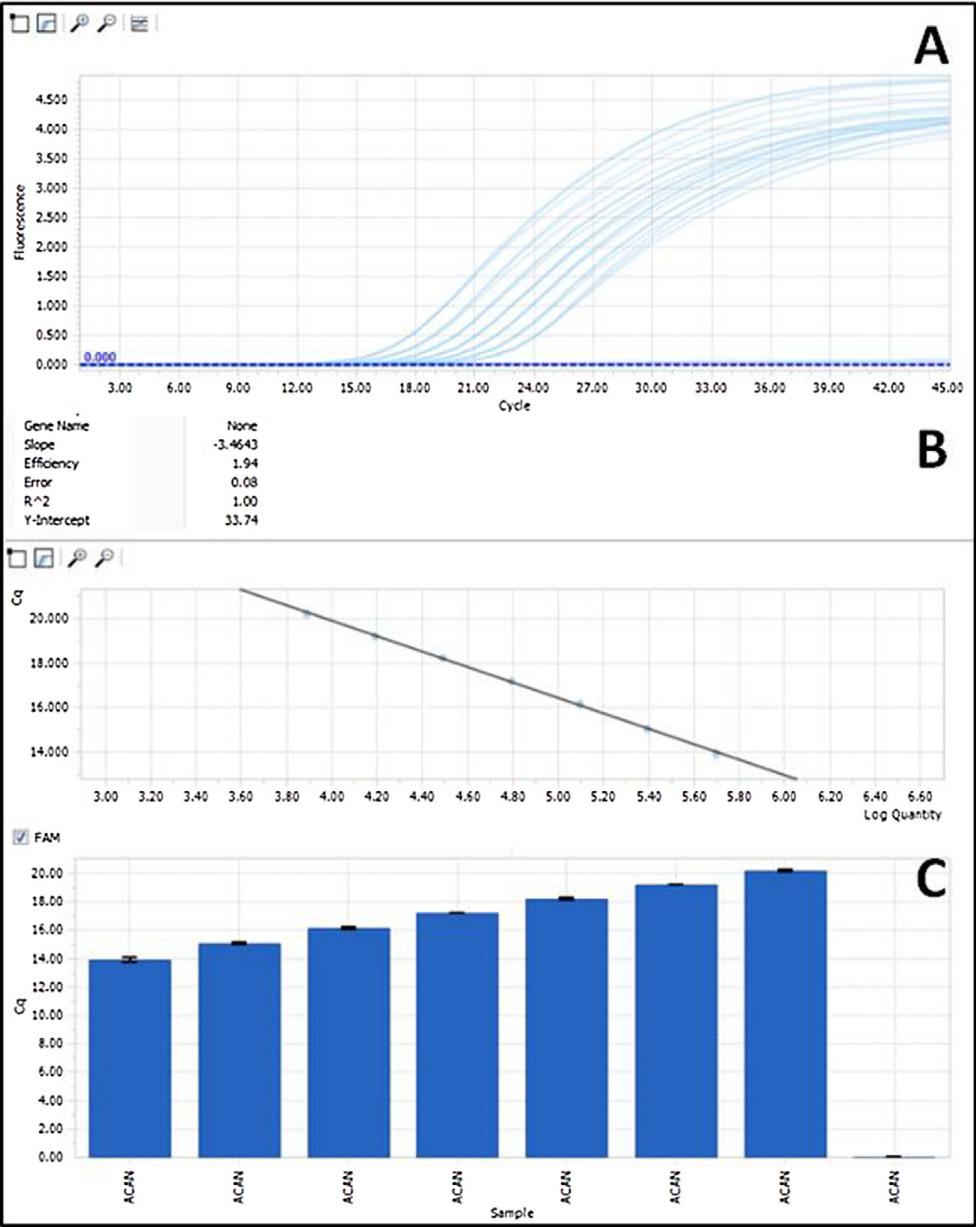
Example of the efficiency calculation for the gene ACAN.
12. For the used genes the efficiencies and other parameters calculated from the software are shown in Table 3

**Table 3:** Efficiency, Slope, and Error for the used genes.

Gene	Efficiency	Slope	Error
GAPDH	1.97	−3.3986	0.07
COL2A1	1.92	−3.5212	0.04
COL1A1	1.95	−3.4501	0.10
ACAN	1.94	−3.4643	0.08
MMP-1	2.02	−3.2646	0.13
MMP-13	2.02	−3.2657	0.16

**Table 4:** Parameters for the bovine primer and probes design.

NM_001034034 (GAPDH)												
		Positions								Dimer		
		Mature Translation			Transcript					ΔG (kcal/mol)		
	Strand	Start	End	Length	Start	End	T_M_ (°C)	GC (%)	Hairpin	Forward	Reverse	Probe
Forward	+	26	46	21	26	46	61.9	47.6	0.00	−5.44	−3.61	−3.47
Probe	−	98	75	24	2164	354	67.8	54.2	0.00			−5.05
Reverse	−	158	140	19	2224	2206	62.4	52.6	0.00		−2.90	−5.05

NM_001001135 (COL2A1)												
		Positions								Dimer		
		Mature Translation			Transcript					ΔG (kcal/mol)		
	Strand	Start	End	Length	Start	End	T_M_ (°C)	GC (%)	Hairpin	Forward	Reverse	Probe
Forward	+	4075	4094	20	15689	15708	62.0	50.0	0.00	−5.16	−5.30	−4.96
Probe	+	4112	4135	20	15726	16038	68.0	58.3	−0.40			−6.58
Reverse	−	4223	4204	20	16126	16107	61.7	55.0	0.00		−3.44	−4.57

NM_001034039 (COL1A1)												
		Positions								Dimer		
		Mature Translation			Transcript					ΔG (kcal/mol)		
	Strand	Start	End	Length	Start	End	T_M_ (°C)	GC (%)	Hairpin	Forward	Reverse	Probe
Forward	+	4075	4094	20	15689	15708	62.0	50.0	0.00	−5.16	−5.30	−4.96
Probe	+	4112	4135	20	15726	16038	68.0	58.3	−0.40			−6.58
Reverse	−	4223	4204	20	16126	16107	61.7	55.0	0.00		−3.44	−4.57

NM_173981 (ACAN)												
		Positions								Dimer		
		Mature Translation			Transcript					ΔG (kcal/mol)		
	Strand	Start	End	Length	Start	End	T_M_ (°C)	GC (%)	Hairpin	Forward	Reverse	Probe
Forward	+	2083	2104	22	40099	40120	62.8	50.0	0.00	−4.34	−3.68	−4.56
Probe	−	2161	2140	22	41252	41231	68.1	59.1	−0.63			−5.46
Reverse	−	2201	2182	20	41292	41273	62.7	50.0	0.00		−2.60	−4.54

NM_174112 (MMP-1)												
		Positions								Dimer		
		Mature Translation			Transcript					ΔG (kcal/mol)		
	Strand	Start	End	Length	Start	End	T_M_ (°C)	GC (%)	Hairpin	Forward	Reverse	Probe
Forward	+	565	584	20	1616	1635	61.9	50.0	−0.97	−6.56	−5.58	−4.97
Probe	−	671	651	21	2725	2705	68.2	57.1	0.00			−6.65
Reverse	−	713	693	21	2767	2747	62.0	47.6	0.00		−2.90	−4.18

NM_174389 (MMP-13)												
		Positions								Dimer		
		Mature Translation			Transcript					ΔG (kcal/mol)		
	Strand	Start	End	Length	Start	End	T_M_ (°C)	GC (%)	Hairpin	Forward	Reverse	Probe
Forward	+	824	844	21	5357	5377	61.7	47.6	0.00	−2.56	−2.73	−3.63
Probe	+	866	890	25	5399	5423	68.0	56.0	0.00			−4.66
Reverse	−	961	943	19	6476	6458	62.1	52.6	−0.73		−7.04	−5.99
